# Facile In Situ Building of Sulfonated SiO_2_ Coating on Porous Skeletons of Lithium-Ion Battery Separators

**DOI:** 10.3390/polym16182659

**Published:** 2024-09-20

**Authors:** Lei Ding, Dandan Li, Sihang Zhang, Yuanjie Zhang, Shuyue Zhao, Fanghui Du, Feng Yang

**Affiliations:** 1 Shandong Key Laboratory of Chemical Energy Storage and New Battery Technology, School of Chemistry and Chemical Engineering, Liaocheng University, No. 1, Hunan Road, Liaocheng 252000, China; dinglei921022@163.com (L.D.); lidandan10224@163.com (D.L.); zhshuyue09@163.com (S.Z.); dufanghui@lcu.edu.cn (F.D.); 2 School of Food Science and Engineering, Hainan University, 58 Renmin Avenue, Haikou 570228, China; 3 Department of Chemistry and Biology, Liaocheng University Dongchang College, No. 266, North Outer Ring Road, Liaocheng 252001, China; yuanjiezhang2016@163.com; 4 State Key Laboratory of Polymer Materials Engineering, College of Polymer Science and Engineering, Sichuan University, No.24 South Section 1, Yihuan Road, Chengdu 610065, China; yangfengscu@126.com

**Keywords:** lithium-ion battery separator, sulfonated SiO_2_, in situ coating, pore size dispersion, cycling stability

## Abstract

Polyolefin separators with worse porous structures and compatibilities mismatch the internal environment and deteriorate lithium-ion battery (LIB) combination properties. In this study, a sulfonated SiO_2_ (SSD) composited polypropylene separator (PP@SSD) is prepared to homogenize pore sizes and in situ-built SSD coatings on porous skeletons. Imported SSD uniformizes pore sizes owing to centralized interface distributions within casting films. Meanwhile, abundant cavitations enable the in situ SSD coating to facilely fix onto porous skeleton surfaces during separator fabrications, which feature simple techniques, low cost, environmental friendliness, and the capability for continuous fabrications. A sturdy SSD coating on the porous skeleton confines thermal shrinkages and offers a superior safety guarantee for LIBs. The abundant sulfonic acid groups of SSD endow PP@SSD with excellent electrolyte affinity, which lowers Li^+^ transfer barriers and optimizes interfacial compatibility. Therefore, assembled LIBs give the optimal C-rate capacity and cycling stability, holding a capacity retention of 82.7% after the 400th cycle at 0.5 C.

## 1. Introduction

Owing to the features of low self-discharge, excellent safety performance, stable cycle life, and high energy density, lithium-ion batteries (LIBs) have been widely used in energy storage devices including portable electronic devices and electric vehicles for more than 30 years since their first commercialization [1,2]. Four components of anode, cathode, separator, and electrolyte amalgamate to combine into LIBs. Separators are thus seated between two electrodes to avoid the physical contact of electrodes and meanwhile conduct Li^+^, which requires separators to possess both absolute insulation and suitable porous structures [3,4]. Especially, porous structures govern ion transfer paths and affect battery performance even though chemically inert separators are not involved in electrode redox reactions [5]. Even and centralized pore size can ensure homogenized Li^+^ flux and effectively stabilize the separator/electrode interface process [6,7]. Furthermore, features like flexibility, sufficient mechanical strength, low manufacturing cost, and electrolyte affinity are also vital for separators. Hence, separators prepared with polyolefin have become mainstream in the separator market nowadays [8,9].

However, the low surface energy of polyolefins generates instinctive hydrophobic properties and inevitably deteriorates electrolyte affinity. Since ions only migrate within the electrolyte-filled pores, poor electrolyte affinity determines partially lacking filling conditions and increases ion migration barriers [10,11,12]. Also, routine polyolefins own low melting points of about 130 °C and 165 °C for polyethylene and polypropylene. This limits weak thermal stability and tends to contract excessively at elevated temperatures, which increases the risk of short circuits caused by electrode contacts [13,14]. However, regular LIB operations produce massive heat and raise inner temperatures. The poor dimensional tolerance of separators to heating thus cannot ensure security for LIBs [15,16].

Building inorganic nano-ceramic layers, such as TiO_2_, Al_2_O_3_, SiO_2_, and ZrO_2_ with various functionalizations, on separator top surfaces can construct sturdy skin skeletons to not only intensify electrolyte affinity but enhance thermal stability in actual separator industrial fabrications [17,18,19,20,21,22,23,24,25,26]. However, the matched binders are needed to ensure tight adhesions between surface coating and separators. Excess binders may seep into interstitial paths of nano-ceramics and separator surface pores, which chokes partial ion migration routes and minifies electrochemical performance. The superficial nano-ceramic coatings necessarily increase extra thickness and reduce battery energy density [27,28]. Since the nanoparticle coatings only appear on separator top surfaces, the electrolyte wettability and ion migration process within separator inner porous skeletons cannot be optimized [29,30,31,32]. In particular, existing research and commercial separators are coated offline. The inorganic nanoparticle coating is elaborately spread on the separator top surface after separator preparations or porous structure formations, which elevates facility costs and technological difficulty. Therefore, exploring a new coating separator manufacturing process to achieve the simultaneous in situ construction of ceramic coatings on the porous skeletons during pore formations is critical for the low-cost manufacturing of high-performance separators.

Presently, actual industrial separator manufacturing processes including dry processes and wet processes are based on the tensile-induced pore formation of casting films [33,34,35,36]. The dry process can be divided into uniaxial tensile and biaxial tensile. The wet process adopts casting film containing ultra-high molecular weight polyethylene and mineral oil and exerts biaxial drawing and solvent extraction to obtain a porous skeleton. The dry process, with uniaxial drawing, inflicts an ultra-high die draw ratio during extrusion, then undergoes annealing and uniaxial drawing to create acerose pores. Particularly, the dry process with biaxial drawing (DPBD) applies directly tensile on casting sheets, in which the β-crystal polypropylene lamellae within sheets are forced to separate due to incompact arrangements and further evolve into the final porous structure under biaxial drawing. Compared with other separator preparation processes, DPBD omits organic solvent extractions and annealing treatment and thus features eco-friendly, low-cost, and continuous productions, which owns tremendous potential for separator production [37,38]. However, the unique pore-forming process determines the scattered pore size [39,40] since lamellae with heterogeneous distribution present various deformation patterns, resulting in plentiful remaining coarse fibrils and worsening cavitation effects [41,42,43]. Blending nano-ceramics into β-crystal polypropylene casting sheets can improve interface distributions and thus visibly thin coarse fibrils owing to the stripping between nano-ceramics and lamellae. Meanwhile, ample cavities stem from direct separations of ceramic/lamellae interfaces and thus facilely fix the ceramic on the porous skeleton surface, with features including simple techniques, low cost, environmental friendliness, and the capability for continuous fabrications.

With these considerations in mind, nanometer-sized SiO_2_ was first sulfonated (SSD) to optimize hydrophilicity in this research. Then, SSD-composited separators (PP@SSD) were prepared based on the DPBD to simultaneously homogenize pore sizes and in situ build SSD coatings on porous skeleton surfaces, featuring simple techniques, low cost, environmental friendliness, and the capability for continuous fabrications. Furthermore, the PP@SSD separator presented optimized thermal stability owing to the robust SSD coating on the porous skeleton. Abundant sulfonic acid groups on the SSD coating further endowed electrolyte affinity to the separator surface and inner pore walls, lowered Li+ transfer barriers, and optimized interfacial compatibility. Consequently, the PP@SSD separator gave assembled LIBs the optimal C-rate capacity and cycling stability. This approach offered practical guidance for the low-cost mass manufacturing of high-performance separators.

## 2. Experiments

### 2.1. Separator Preparation

An amount of 4 g of nano-SiO_2_ (Hydrophilic-200, 7–40 nm, Shanghai Aladdin Biochemical Technology Co., Ltd., Shanghai, China) was added into a mixed solution containing 100 mL methanol and 2 mol concentrated sulfuric acid. The mixed solution successively experienced ultrasonic concussion for 100 min, suction filtration, vacuum drying at 100 °C for 24 h, and then trituration to obtain sulfonated SiO_2_ (SSD). A casting film containing 10 wt% SSD, 89.7 wt% polypropylene (S801, Korea Petrochemical Ind. Co., Ltd., Shanghai, China), and 0.3 wt% β-crystal nucleating agent (NAB83, GCH Technology Co. Ltd., Guangzhou, China) was prepared based on the double-screw extruder (die temperature: 210 °C, casting temperature: 125 °C). Then, biaxial drawing was applied on casting films based on the Bruckner KARO IV biaxial stretcher (Bruckner Group, Siegsdorf, Germany) to prepare a porous PP@SSD separator, which contained the longitudinal drawing along the MD (drawing ratio: 300%, drawing temperature: 100 °C) and sequential drawing vertical to the MD (namely TD, drawing ratio: 300%, drawing temperature: 120 °C). For comparison, untreated SiO_2_ (SD) and β-crystal polypropylene were composited to, respectively, produce pure PP and PP@SD separator.

### 2.2. Tests and Characterizations

The crystal morphologies of the casting films and the porous skeleton morphologies of the separators were observed by FEI Inspect F scanning electron microscopy (SEM). Before testing, casting films were first etched by the mixed acid (H_3_PO_4_:H_2_SO_4_ = 1:1, volume ratio, the concentrations of H_2_SO_4_ and H_3_PO_4_ used in this research are 85% and 98.3%) containing 1.5 wt% KMnO_4_ for 24 h to remove the amorphous portion. The melting behaviors of casting films and separators are recorded by Mettler Toledo DSC3+ Differential scanning calorimetry (DSC, 25–210 °C, 20 °C/min). The DSC crystallinity (X_C,DSC_) could be obtained based on the ratio of the sample melting enthalpy to that of 100% crystalline polypropylene. The DSC β-crystal content (K_β,DSC_) was calculated by the ratio of the β-crystal crystallinity to X_C, DSC_. The thermal stability of casting films was assessed on the Q-500 thermogravimetric (TG) analysis (30–800 °C, 15 °C/min). A D8 ADVANCE X-ray diffractometer (XRD) was used to gain XRD spectra. The XRD crystallinity (X_C,XRD_) was obtained by calculating the diffraction area fractions of the crystalline phase. The XRD β-crystal content (K_β,XRD_) was the area ratio of the β-phase to crystalline regions. 

Separator porosity was acquired by (V_T_ − V_S_ − V_P_)/V_T_, where V_T_ is the total volume of tested separators, and V_S_ and V_P_ are volumes occupied by SSD and polypropylene. Gurley values were measured on the 4110N Gurley tester. The contact angle was tested on the KRUSS K100 (KRÜSS Scientific Instruments Co., Ltd., Hamburg, Germany) contact angle meter. Electrolyte uptake was calculated by (W_a_ − W_i_)/W_i_, where W_i_ is the initial separator weight and W_a_ is the separator weight after immersion in ethylene carbonate (EC): diethyl carbonate (DEC) = 1:1 (mass ratio) for 6 h. Electrolyte retention was obtained by (W_r_ − W_i_)/(W_a_ − W_i_), where W_r_ is the weight of the separator placed in a sealed electronic scale at various times. Tensile and puncture properties were measured by a universal testing machine fitted with a heating chamber. The thermal shrinkage conditions of separators can be calculated by A_c_/A_i_, where A_i_ is the initial area of the separators, and A_c_ is the contractive separator area after being subjected to various temperatures for 30 min (measured by open source software “Image J”, version number: 1.52).

A Reference 3000 (Gamry Instruments, Warminster, PA, USA) electrochemical workstation was used to measure separator electrochemical performance. The electrochemical stability window was recorded by the linear sweep voltammetry (LSV) of the Li-stainless steel (SS) cell (2–6 V, 5 mV/s). The alternating-current (AC) impedance of the SS/SS cell (10^6^–10^−2^ Hz, 10 mV) was recorded to obtain bulk impedance (R_b_). Ionic conductivity (σ) was thus measured by σ = T/(R_b_·S), where T and S are the thickness and area of separators. Ion migration activation energy (E_a_) was measured by σ = σ_0_·exp(−E_a_/RT), where σ_0_, R, and T are the pre-factor, thermodynamic gas constant, and temperatures. Interface impedance (R_i_) was assessed by the AC impedance of the Li/Li cell. Li^+^ transfer number (t_+_) was managed by t_+_ = I_s_(ΔV − I_i_R_i_)/I_i_ (ΔV − I_s_R_s_), where ΔV is the chronoamperometry step potential (10 mV), R_s_ and I_s_ are the steady impedance and current on chronoamperometry plots, and I_i_ is the initial current of chronoamperometry. A CT3001A LAND (Wuhan LAND Electronic Co. Ltd., Wuhan, China) system was used to evaluate the C-rate discharge capacity retention and long-term effective cycling life of LiCoO_2_/Li cells assembled with three separators (constant temperature conditions at 26 °C, voltage range: 2.75–4.25 V). The C-rate capacities of LiCoO_2_/Li cells were recorded under the charge current of 0.2 C and discharge conditions of 0.2, 0.5, 1, 2, 4, and 8 C, respectively. Cycling tests were carried out at a constant charge–discharge current of 0.5 C. After assembly, the LiCoO_2_/Li cells were left to set for 12 h and following activation (cycled three times at 0.1 C). Customized LiCoO_2_ cathodes with 95.7 wt% active material, SS foil, lithium foil, and electrolyte (1 M LiPF_6_ dissolved in solvents composed of EC, DEC, and dimethyl carbonate (DMC)) were offered by Kejing Material Technology Co., Ltd., Shenzhen, China.

## 3. Results and Discussion

### 3.1. Properties of Casting Films

**Figure 1:**
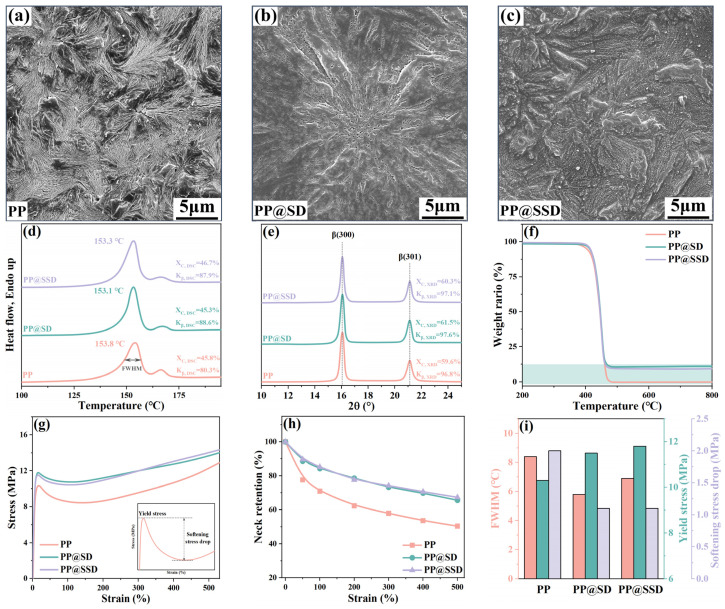
SEM images of three casting films: (**a**) PP, (**b**) PP@SD, and (**c**) PP@SSD. Key properties of casting films: (**d**) DSC heating scans, (**e**) XRD spectra, (**f**) TG plots, (**g**) drawing curves, (**h**) neck retention during drawing. (**i**) Crucial parameters of casting films (FWHM: full width at half maximum of β-phase melting peak, indicated in (**d**)).

The crystal morphology SEM images of three casting films are listed in Figure 1a–c. Abundant β-lamellae with a loose arrangement can be detected for the pure PP casting film, without any compact α-phase features. The XRD spectra of the PP film in Figure 1e present X_C,XRD_ of 59.6% and high K_β,XRD_ of 96.8%, which indicates pure β-lamellae in the PP film. Melt-recrystallizations of metastable β-phase at elevated temperatures generate the primary β-phase endothermic peak at 153.3 °C and inapparent α-phase melting peak at 166.4 °C on DSC heating scans (Figure 1d). Since XRD spectra show almost 100% K_β,XRD_ content, the lowest K_β,DSC_ (80.3%) of the PP film indicates the inferior β-lamellae thermal stability. Normal SD and sulfonated SSD are uniformly distributed between β-lamellae in the PP@SD (Figure 1b) and PP@SSD (Figure 1c) casting films. Tagged diffraction signals of β-phase (300) and (301) lattice planes at 16.1° and 21.2° appear on XRD spectra, together with the high K_β,DSC_ of 97% and invisible α-phase diffraction signals, manifesting the undeteriorated β-phase after SD and SSD additions. TG heating plots (Figure 1f) display similar initial decomposition temperatures of about 420 °C for three films but higher weight retentions for PP@SD (10.5%) and PP@SSD (9.7%). The similar melting points of about 153 °C and higher K_β,DSC_ for PP@SD (88.6%) and PP@SSD (87.9%) on DSC heating plots signify a more stable β-phase. Furthermore, the PP film presents the wide full width at half maximum of the β-phase melting peak (FWHM, 8.4 °C). Meanwhile, the narrower FWHMs of PP@SD (5.8 °C) and PP@SSD (6.7 °C) reflect centralized β-lamellae distributions, which can homogenize the deformation manners of casting films and consequent porous structure. The above mitigatory deformations can be proved by the gently drawing curves of PP@SD and PP@SSD (Figure 1g, drawing rate: 100 mm/min, drawing temperature: 100 °C). Profiting from the reinforcement of SSD, PP@SSD shows a higher yield stress of 11.8 MPa. Also, numerous interfaces between β-lamellae and SSD improve interface distributions and thus reduce stress concentration during the drawing process, exhibiting a smaller softening stress drop of 1.1 MPa and higher neck retentions for PP@SSD (Figure 1h,i).

### 3.2. Porous Structures

**Figure 2:**
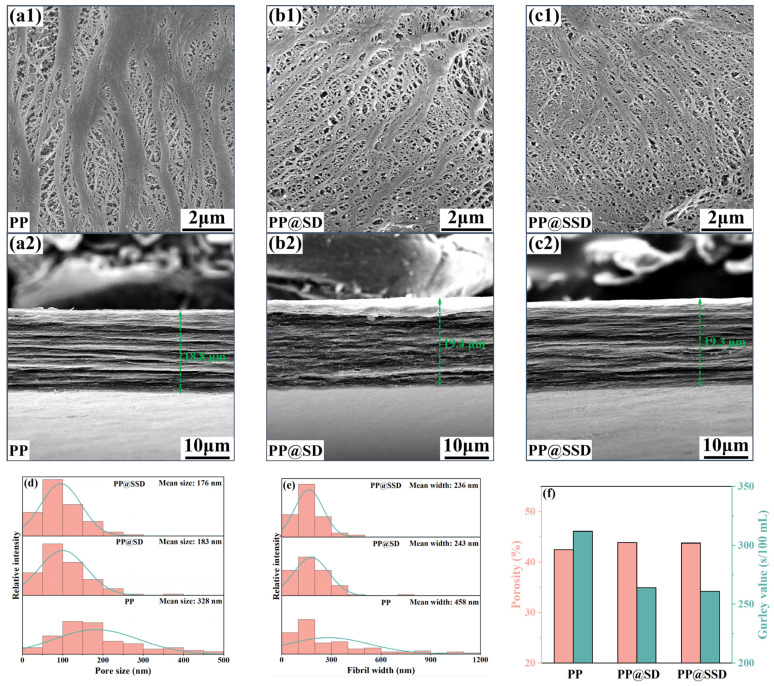
SEM images of three separators: (**a**) PP, (**b**) PP@SD, and (**c**) PP@SSD (1: surface morphology; 2: cross-section morphology). (**d**) Pore size distributions of separators. (**e**) Fibril width distributions of separators. (**f**) Porosity and Gurley value of separators.

The separator morphologies (containing surface and cross-section) after the biaxial drawing are depicted in Figure 2a–c to verify the effects of the SSD on the porous structure. Pore size and fibril width distributions are listed in Figure 2d,e, accompanied by the porosity and Gurley value in Figure 2f. Longitudinal drawing compels β-lamellae along the TD to separate, while compacting β-lamellae along the MD to transform into dense α-phase fibrils. Subsequent transverse drawing can expand pore size but still retain numerous coarse fibrils on the surface. The above heterogeneous deformation modes thus generate obviously fractured fibrils and uneven pore size for the PP separator, which can be further proved by the broad pore size (mean size: 328 nm) and fibril width (mean width: 458 nm) distributions. Furthermore, the PP separator exhibits the typical multilayer stacked structure with a thickness of 18.8 µm due to the plane biaxial drawing. A normal porosity (42.5%) and a high Gurley value (312 s/100 mL) of the PP separator also signify the worse permeability. SD and SSD optimize interface distribution within casting films and relieve stress concentrations during drawing. Even if coarse fibrils emerge in the longitudinal drawing, the SD(SSD)/PP interfaces can be stripped again in the following transverse drawing, visibly narrowing fibril widths and concentrating pore size distribution. Consequently, PP@SD and PP@SSD present uniform porous structures, together with similar mean pore sizes of 183 and 176 nm. The remarkable coarse fibrils cannot be detected on the surfaces of two separators. Average fibril widths also reduce to 236 and 243 nm, respectively. The higher porosity (PP@SD: 43.9%, PP@SSD: 43.8%) and lower Gurley value (PP@SD: 263 s/100 mL, PP@SSD: 261 s/100 mL) further certify the superior pore channel linearity, which can provide smoother paths for ion migrations.

### 3.3. Wettability, Thermal Stability, and Mechanical Properties

**Figure 3:**
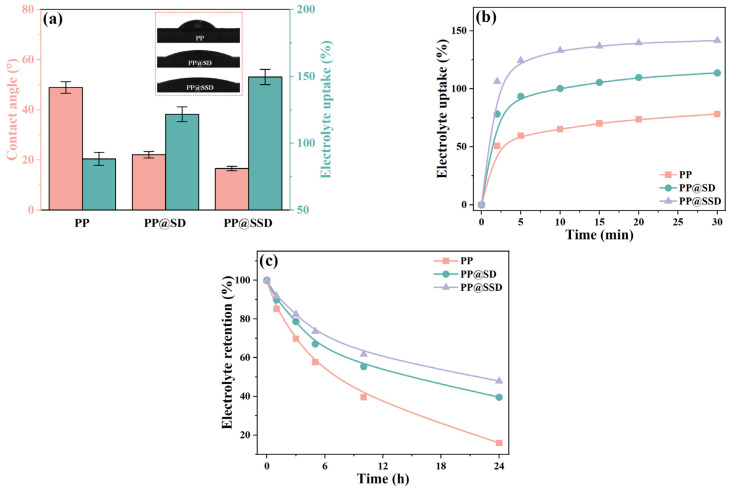
(**a**) Contact angle and electrolyte uptake of three separators. (**b**) Electrolyte uptake at various immersion times. (**c**) Electrolyte retention after immersion.

Better compatibility between separators and electrolytes improves electrolyte-filling processes into porous structures and reduces ion migration barriers [44,45,46]. The characteristic contact angle and electrolyte uptake are exhibited in Figure 3a. The PP separator shows a contact angle of 48.9° and electrolyte uptake of 88.3% because the low surface energy feature of polypropylene deteriorates electrolyte affinity. PP@SD and PP@SSD give superior electrolyte wettability owing to the SD and SSD coating on porous skeleton surfaces and thus reduce contact angles to 22.1° and 16.6°, maybe due to the transition from the Cassie–Baxter state to the Wenzel state. The stronger capillary intrusions further reinforce electrolyte uptake to 121.7% for PP@SD and reach a maximum of 149.6% for PP@SSD. Stronger capillary intrusions also endow PP@SSD with the fastest electrolyte absorption speed (Figure 3b), achieving electrolyte uptake of 133.1% after immersion for only 10 min. Furthermore, the highest electrolyte retention of PP@SSD (Figure 3c) demonstrates the best electrolyte retention capacity owing to the abundant sulfonic acid group.

**Figure 4:**
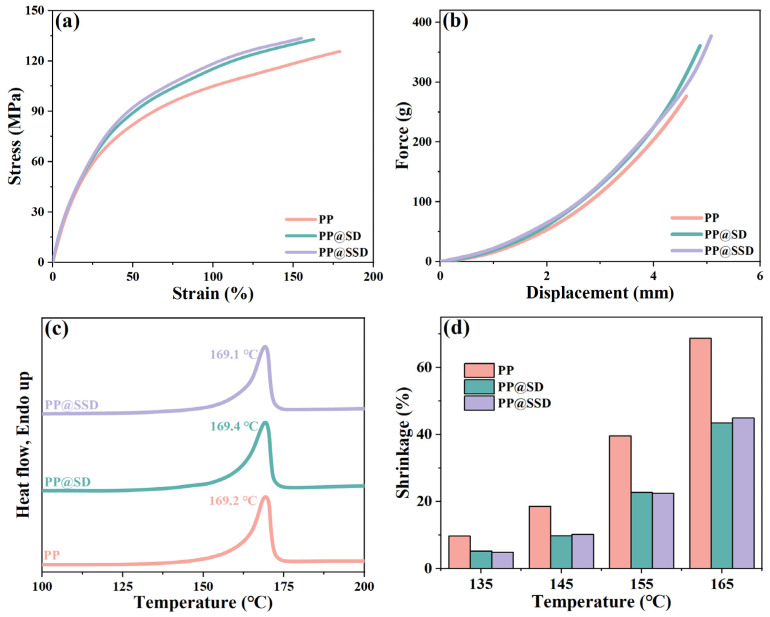
(**a**) Tensile and (**b**) puncture plots of separators. (**c**) DSC heating curves of separators. (**d**) Thermal shrinkage conditions at various temperatures.

Separators encounter various conditions including tensile, coiling, compression, and puncture during LIB assembly and subsequent long-term operations [47,48]. Sufficient strengths are necessary for guaranteeing separator integrality. The tensile and puncture plots are listed in Figure 4a,b (drawing and puncture rate: 200 mm/min, temperature: 25 °C). The PP separator shows the drawing and puncture strength of 125.7 MPa and 276.5 g, which can cope with various encounters faced within LIBs. PP@SD and PP@SSD give a high drawing strength of 132.9 and 133.6 MPa owing to nanoparticle reinforcement effects. Meanwhile, the much higher puncture strength for PP@SD (360.8 g) and PP@SSD (377.2 g) can provide utilization potentiality for LIBs with high-security demands. 

Stable thermal behaviors of separators act to prevent short circuits as LIBs are faced with thermal runaways. The DSC plots and thermal shrinkage states at various temperatures are displayed in Figure 4c,d. Since the metastable β-lamellae spontaneously translate into the α-phase under tensile and thermal stimuli, three separators show similar α-phase melting behaviors (PP: 169.1 °C, PP@SD: 169.4 °C, and PP@SSD: 169.2 °C), whereas the normal PP separator exhibits drastic thermal shrinkage of 68.7% at 165 °C. The contractions of PP@SD and PP@SSD are alleviated at elevated temperatures owing to the in situ SD and SSD coating on the porous skeleton surfaces, reaching 43.4% and 44.9% at 165 °C, respectively.

### 3.4. Electrochemical Performance

**Figure 5:**
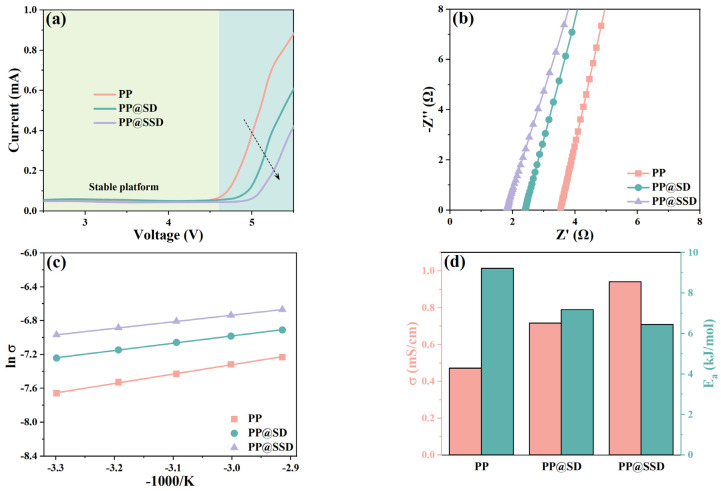

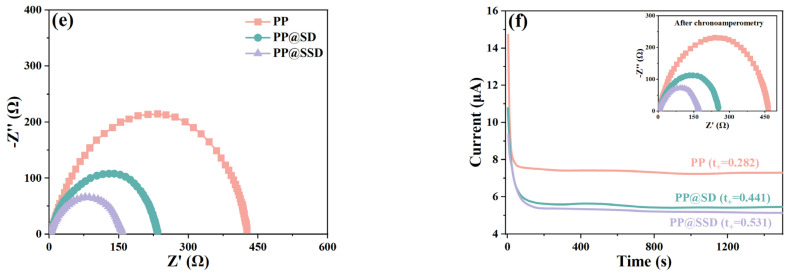
(**a**) LSV curves of Li/SS cells with separators. (**b**) AC impedance plots of SS symmetric cells. (**c**) Arrhenius plots as the temperature rises. (**d**) Ionic conductivity (σ) and activation energy (E_a_). (**e**) AC impedance plots of Li symmetric cells. (**f**) Chronoamperometry curves and calculated Li^+^ transfer number (t_+_) of three separators.

The LSV curves of Li/SS cells containing separators are presented in Figure 5a to evaluate separator electrochemical stability windows. Three separators show placid voltage platforms within 4.6 V, followed by the subsequent current increases owing to electrolyte decompositions. The PP separator maintains stable voltages until 4.73 V. PP@SD shows a stable voltage limit of 4.93 V. The highest stable voltage of 5.08 V and minimum current uncover the superior electrolyte stabilizing for PP@SSD, which can endow separators with more adaptable capacity to high power density LIBs and even the expectant lithium-metal batteries.

Figure 5b shows the AC impedance curves of SS symmetric cells, accompanied by the calculated ionic conductivity (σ) in Figure 5d. The ions can only be compelled to transfer by using electrolytes as carriers owing to the insulating polypropylene substrate. σ is thus mainly governed by the intrinsic pore channel linearity of separators and electrolyte filling conditions within the porous structure. The PP separator exhibits an impedance of 3.54 Ω and σ of 0.47 mS/cm. The SD coating on the porous skeleton optimizes electrolyte affinity and permeability, which endow PP@SD with an impedance of 2.39 Ω and a higher σ of 0.72 mS/cm. Sulfonated SSD coating with an abundant sulfonic acid group owns the optimal hydrophily and thus improves σ to 0.94 mS/cm. Furthermore, in order to assess the ion removability facilitated by the separators, ion transfer activation energy (E_a_) is further calculated by the Arrhenius formula in Figure 5c,d [49]. The high E_a_ of 9.21 kJ/mol for the PP separator signifies the high ion migration barriers. The PP@SD separator with SD coating gives a lower E_a_ of 7.18 kJ/mol, while the lowest E_a_ of 6.45 kJ/mol reveals the highest ion mobility within the PP@SSD separator with SSD coating.

The σ of separators represents the overall ion migration capacity within separators owing to the dual-ion conductions of LIBs, whereas the migration of anion inevitably generates electrode polarizations and sets off chain side reactions. Hence, the Li^+^ transfer number (t_+_) is obtained based on the chronoamperometry to evaluate the current proportion contributed by only Li^+^ migrations [50]. The weak electrolyte affinity of polyolefin separators generates scant contact between porous channels and electrolytes and thus elongates ion migration routes in reverse. The PP separator with inferior wettability thus gives the low-level t_+_ of 0.282. While superior electrolyte affinity can improve electrolyte filling and contact conditions within pore channels, the luxuriant hydroxyl groups on SD surfaces optimize electrolyte compatibility. Meanwhile, the lone pairs in hydroxyl electrostatically interact with Li^+^, which accelerates Li^+^ desolvation and raises free ion concentrations [51]. Furthermore, hydrogen bonds form between PF_6_^−^ and hydroxyl, which hinders anion transfer within separators. Consequently, PP@SD exhibits the t_+_ of 0.441. The highest t_+_ of PP@SSD (0.531) indicates that sulfonic acid groups are significantly more advantageous in promoting Li^+^ transport compared with hydroxyl, which is consistent with the calculated E_a_.

The AC impedance curves of Li/Li cells are displayed in Figure 5e to compare the interfacial impedances (R_i_) of three separators, which state the compatibility between the separator and Li electrode [52]. The high R_i_ value of 426 Ω for the PP separator reflects poor compatibility with Li electrodes. Better electrolyte uptake and retention enable sufficient contact with the Li electrode and improve more suitable interfaces, leading to the much lower R_i_ of 233 and 158 Ω for PP@SD and PP@SSD.

### 3.5. Battery Performance

**Figure 6:**
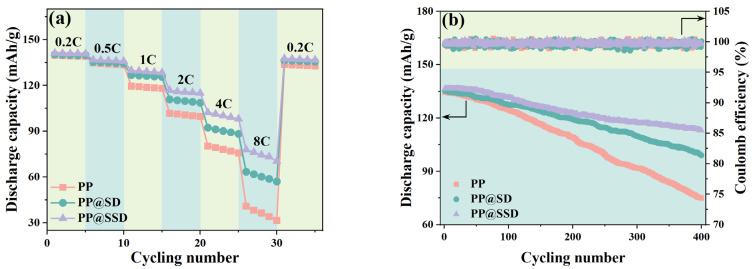
(**a**) C-rate capacity and (**b**) cycling stability of assembled LiCoO_2_/Li cells with three separators.

The C-rate capacities of LiCoO_2_/Li cells with three separators are shown in Figure 6a to testify new-type separator-determined battery combination performance. Cells containing the PP separator show a discharge capacity of about 139–140 mA h/g when cycling at 0.2 C for the first five charge–discharge processes. The limited ion migrations caused by ohmic polarization significantly reduce discharge capacities at the higher current densities, which decrease to 40.9 mA h/g when cycling once at 8 C and reach the minimum of 31.6 mA h/g only after the 5th cycle. Cells including PP@SD give discharge capacities of 140.3 and 139.9 mA h/g during the 1st and 5th cycles at 0.2 C but maintain higher capacities as the current density rises, dropping to 63.5 mA h/g for the 1st cycle at 8C and 57.1 mA h/g after the 5th cycle. The electrode active materials routinely determine the battery capacity. Separators between two electrodes can also affect Li^+^ transfer channels and the separator/electrode interfacial process, which alters battery dynamics and comprehensive performance. The enhanced electrolyte affinity ensures superior uptake and retention conditions, thus wetting the electrode effectively and facilitating Li^+^ insertions and removals. The optimized σ, E_a_, and t_+_ state the accelerated migration rate of Li^+^ within separators and alleviative anti-anion polarization effects. Also, the superior compatibilities between separators and electrodes promote Li^+^ diffusions through the electrode/separator interfaces. Consequently, PP@SSD cells show the optimal capacity retentions of 77.9 and 70.4 mA h/g for the 1st and 5th cycles at 8 C.

Figure 6b displays the capacity retentions and corresponding coulomb efficiencies of LiCoO_2_/Li cells when cycled at 0.5/0.5 C for 400 cycles to assess the effects of a coated separator on long-term charge–discharge behaviors of LIBs. Similar capacities of 134.5, 135.2, and 136.8 mA h/g of PP, PP@SD, and PP@SSD can be detected for the first cycle, whereas the discharge capacity gaps are highlighted as the cycle increases. The capacities of cells with PP achieve 124.7 mA h/g after the 100th cycle and quickly lower to 75.1 mA h/g (capacity retention: 55.8%) after the 400th cycle. Improved cycling stability emerges for the PP@SD cell, which retains a sluggish capacity decay of 127.5 mA h/g and 99.0 mA h/g after the 100th and 400th cycles. Especially, PP@SSD with sulfonated SSD coating can expedite Li^+^ within cells to where it should migrate, leading to the high capacity during the cycling test and ultimately maintaining a capacity of 113.1 mA h/g (retention: 82.7%).

## 4. Conclusions

In this research, the novel PP@SSD composite separator SSD composited separators were designed based on the DPBD. This approach simultaneously homogenized pore size distributions and fixed to porous skeleton surfaces during PP@SSD separator fabrications, which facilely integrated the ceramic coating during separator fabrications and feature low production difficulty and cost. In addition, the robust SSD coating on porous skeleton surfaces provided superior thermal dimensional stability and adequate security at elevated temperatures. Numerous sulfonic acid groups of the SSD coating also endowed the PP@SSD composite separator with better electrolyte affinity, which lowered barriers for Li^+^ transfer and optimized overall battery performance. This research focused on offering a facile separator manufacturing process, which combines the characteristics of low cost, high security, and high performance for the next generation of LIBs and the expected lithium-metal batteries.

## Data Availability

The original contributions presented in the study are included in the article, further inquiries can be directed to the corresponding author.
